# Folding the unfoldable: using AlphaFold to explore spurious proteins

**DOI:** 10.1093/bioadv/vbab043

**Published:** 2022-01-09

**Authors:** Vivian Monzon, Daniel H Haft, Alex Bateman

**Affiliations:** 1 European Molecular Biology Laboratory, European Bioinformatics Institute (EMBL-EBI), Wellcome Genome Campus, Hinxton CB10 1SD, UK; 2 National Center for Biotechnology Information, National Library of Medicine, Bethesda, MD 20894, USA

## Abstract

**Motivation:**

The release of AlphaFold 2.0 has revolutionized our ability to determine protein structures from sequences. This tool also inadvertently opens up many unanticipated opportunities. In this article, we investigate the AntiFam resource, which contains 250 protein sequence families that we believe to be spurious protein translations. We would not expect proteins belonging to these families to fold into well-ordered globular structures. To test this hypothesis, we have attempted to computationally determine the structure of a representative sequence from all AntiFam 6.0 families.

**Results:**

Although the large majority of families showed no evidence of globular structure, we have identified one example for which a globular structure is predicted. Proteins in this AntiFam entry indeed seem likely to be *bona fide* proteins, based on additional considerations, and thus AlphaFold provides a useful quality control for the AntiFam database. Conversely, known spurious proteins offer useful set of quality controls for AlphaFold. We have identified a trend that the mean structure prediction confidence score pLDDT is higher for shorter sequences. Of the 131 AntiFam representative sequences <100 amino acids in length, AlphaFold predicts a mean pLDDT of 80 or greater for six of them. Thus, particular care should be taken when applying AlphaFold to short protein sequences.

**Availability and implementation:**

The AlphaFold predictions for representative sequences can be found at the following URL: https://drive.google.com/drive/folders/1u9OocRIAabGQn56GljoG1JTDAxjkY1ro.

**Supplementary information:**

Supplementary data are available at *Bioinformatics Advances* online.

## 1 Introduction

Databases of protein sequences, such as UniProt (UniProt Consortium, 2021) and RefSeq (Li *et al.*, 2021), are critical for modern molecular biology. These databases are built upon predictions of protein-coding genes from DNA sequence data. Only a small fraction of these protein-coding gene predictions have support from experimental data. When a gene prediction software makes errors it can lead to the creation of protein sequence entries that are not found in nature. These erroneous protein sequences are what we call spurious proteins. Genome contamination, such as from non-coding repetitive eukaryotic DNA, can exacerbate the problem of erroneously predicted protein-coding sequences (Breitwieser *et al.*, 2019). Spurious proteins account for a small fraction (perhaps 1–2%) of all sequences in the databases. However, due to the size of the databases this still accounts for millions of sequences. It is useful to identify these spurious proteins and remove them from analyses and from biological database resources to improve their accuracy. Over the years a small number of tools and methods have been developed to identify spurious proteins. One of the earliest tools was the AntiFam database (Eberhardt *et al.*, 2012), which contains a collection of profile-HMM models for sets of sequences that are believed to be spurious translations (Eddy, 1998). These profile-HMMs can be used to search any set of sequences of interest. For example, they can be applied to sets of metagenomics sequence predictions to assess the quality of the sequence predictions. Since it was founded, AntiFam has collected a rather modest 250 entries. AntiFam has been curated in a rather *ad hoc* fashion. In its initial period of growth, spurious families were identified in Pfam and removed from that resource and placed into AntiFam. More recently a screen for protein-coding genes that overlapped known non-coding RNA genes, such as tRNAs was carried out and identified proteins clustered to make new entries. Most recently, within release 6.0, we identified proteins, which were found on the opposite strand of known genes. These are the so-called shadow ORFs (open reading frames). This screen contributed the largest number of entries to AntiFam. In this work, we apply AlphaFold to representative sequences from AntiFam to firstly understand how AlphaFold performs on spurious protein sequences, but also to identify if any existing AntiFam entries might actually be *bona fide* proteins with a globular structure.

## 2 Methods

We took the Stockholm formatted seed flatfile for AntiFam release 6.0 with 250 entries and extracted the first representative sequence for each entry and removed all gap characters. We then submitted each sequence for structure determination by the AlphaFold 2.0 software package (Jumper *et al.*, 2021), installed locally, using default settings with May 14, 2020 being the latest template release date (PDB templates available at CASP14) and we used the pTM models. We used the full sequence databases specified by AlphaFold to create the multiple sequence alignments (MSA) for the structure predictions.

To visualize the results, we adapted the code from ColabFold to plot the MSA sequence coverage and the prediction confidence (Mirdita *et al.*, 2021).

To investigate the effect of the sequence length on the AlphaFold prediction results, we randomly created amino acid sequences of different length using the random python library. Each amino acid was weighted equally for selection. We generated five random sequences per sequence length of 20, 30, 40, 50, 60, 70, 80, 90, 100, 120, 140, 160, 180 and 200 residues (Supplementary Table S1). We ran AlphaFold as described above for the AntiFam sequences.

For a direct comparison of spurious proteins with *bona fide* proteins of the SwissProt database (UniProt Consortium, 2021), we ran AlphaFold as described before on five randomly selected sequences of the sequence length 10, 16, 20, 30, 40, 50, 60, 70, 80, 90, 100, 120, 140, 160, 180 and 200. The sequence identifier can be found in Supplementary Table S2. When selecting the sequences, fragmented proteins were avoided and only SwissProt sequences with an average IUPred2A score below 0.5 were selected to avoid disordered proteins (Erdős and Dosztányi, 2020).

For an extended analysis of the AntiFam entries ANF00051, ANF00055, ANF00056, ANF00058, ANF00064 and ANF00208, we used the PSIPRED v. 4.0 web server to predict the secondary structure of each representative sequence (Buchan and Jones, 2019).

## 3 Results

**Fig. 1.**
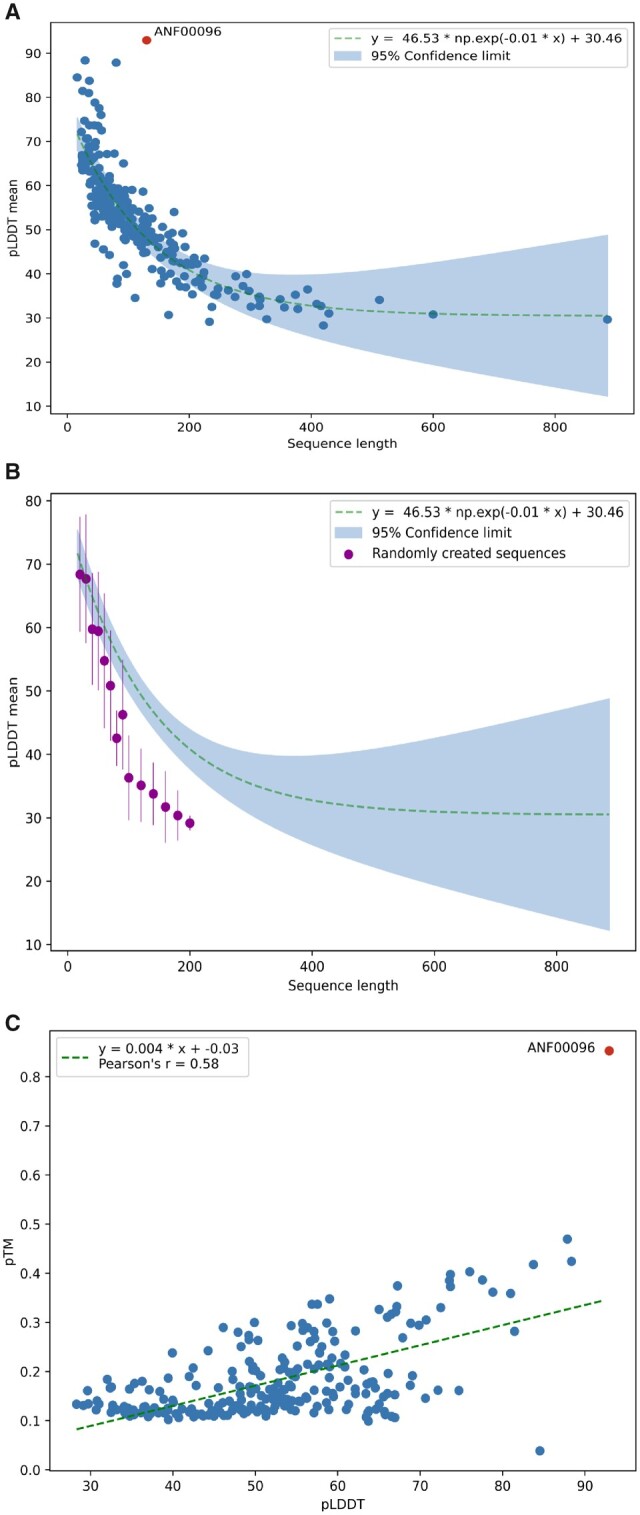
(**A**) Graph showing the relationship between mean pLDDT and sequence length across AntiFam representative sequences. The 95% confidence interval is shown in blue shading. The red dot represents a likely false positive entry in AntiFam (ANF00096). (**B**) Graph showing the relationship between mean pLDDT and sequence length for a range of randomly generated sequences. The confidence interval shown is that calculated for the AntiFam matches in (A). (**C**) In this graph, the pLDDT scores are plotted against the pTM scores for the representative AntiFam sequences with the red dot again representing the likely false positive AntiFam entry (ANF00096)

For each sequence representing an AntiFam family, we computationally determined the structure with AlphaFold 2.0 (Jumper *et al.*, 2021). We manually inspected the results of all 250 predictions. First, we studied the sequence coverage for the AntiFam entries. On average 1233, 851 and 37 homologous sequences were found in the BFD, MGnify and UniRef90 sequence databases, respectively. We observed that the number of homologous sequences found in the BFD database tended to be higher for Antifam entries with a higher sequence length. Second, we inspected the predicted Local Distance Difference Test (pLDDT) plots, which determine whether any region of the sequence has been predicted with high confidence. During this inspection, we noted that many short sequences had a relatively high pLDDT score indicating confident predictions with 17 of the 131 Antifam entries of a sequence length below 100 residues yielding a mean pLDDT above 70. Beyond that, 58 of these 131 short sequence entries had a mean pLDDT above 60 and most of them, 120 of 131 entries, had a mean pLDDT above 50. Only one Antifam entry with a sequence length above 100 residues, ANF00096, yielded a pLDDT score above 70 (see Fig. 1A). The average residue number of the submitted sequences is 126 amino acids ranging from 16 to 886 residues. For further investigation, we plotted the mean pLDDT score of the top ranked AlphaFold prediction for each AntiFam sequence against its length, as shown in Figure 1A. Surprisingly, there is a strong correlation between the mean pLDDT and the length of the sequence, with shorter sequences showing higher mean pLDDT scores. This tendency could be reproduced with randomly generated amino acid sequences of different length (Fig. 1B and Supplementary Table S1) although the slope looks different for the random sequences. The pTM score shows a slight correlation with the pLDDT score, it is below 0.5 for all AntiFam entries except ANF00096 (Fig. 1C).

**Fig. 2.**
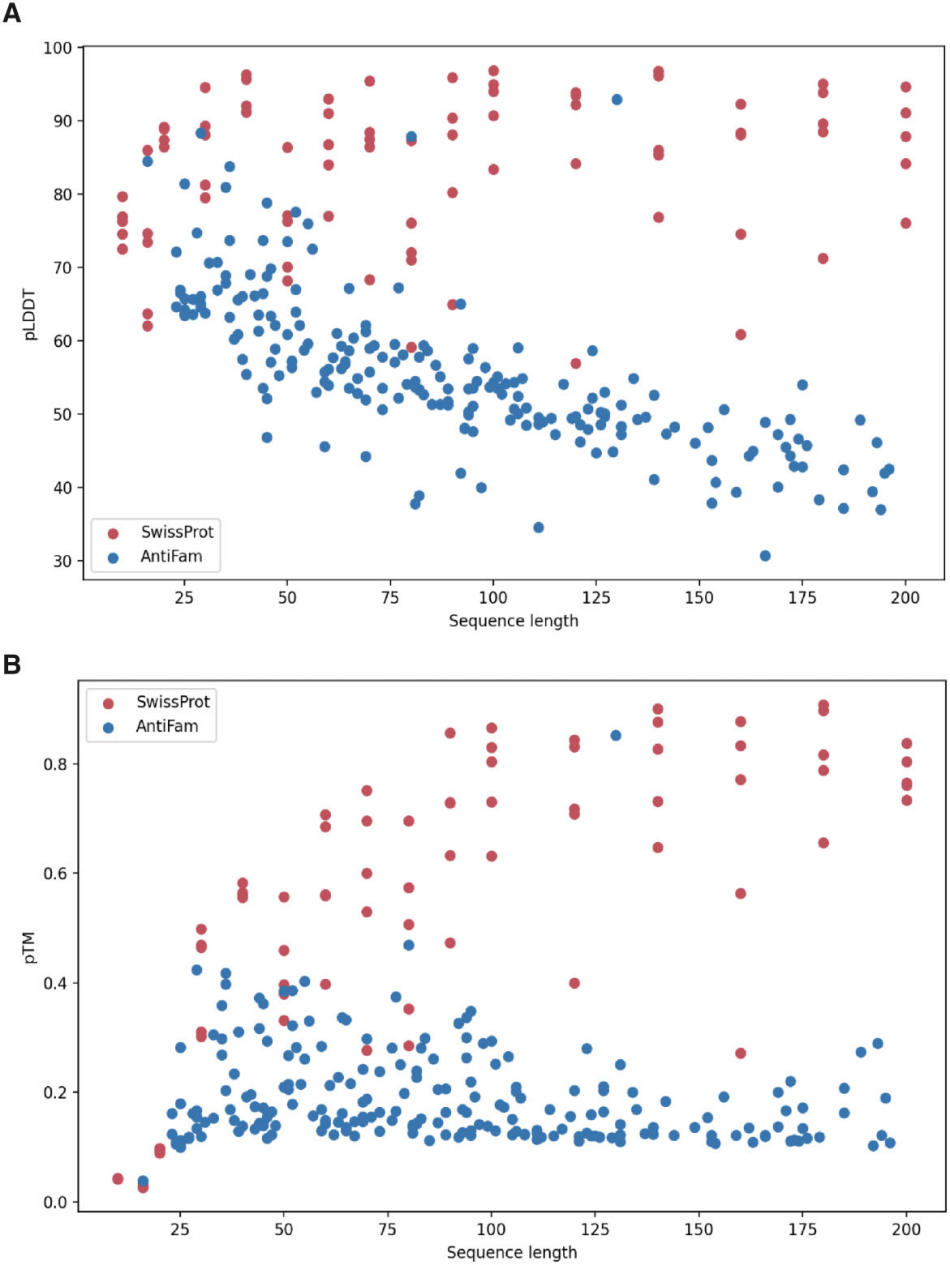
Comparison between spurious AntiFam sequence entries and *bona fide* protein sequences from SwissProt. (**A**) The average pLDDT score against protein sequence length. (**B**) The pTM score against protein sequence length

When comparing AntiFam and SwissProt sequence entries, we noted that the average pLDDT as well as pTM score was higher for SwissProt proteins compared to spurious proteins from a sequence length of around 100 residues, yielding a distinction between spurious and *bona fide* proteins (Fig. 2). For shorter proteins, no clear separation was achieved with either the pLDDT nor with the pTM score (Fig. 2).

**Fig. 3.**
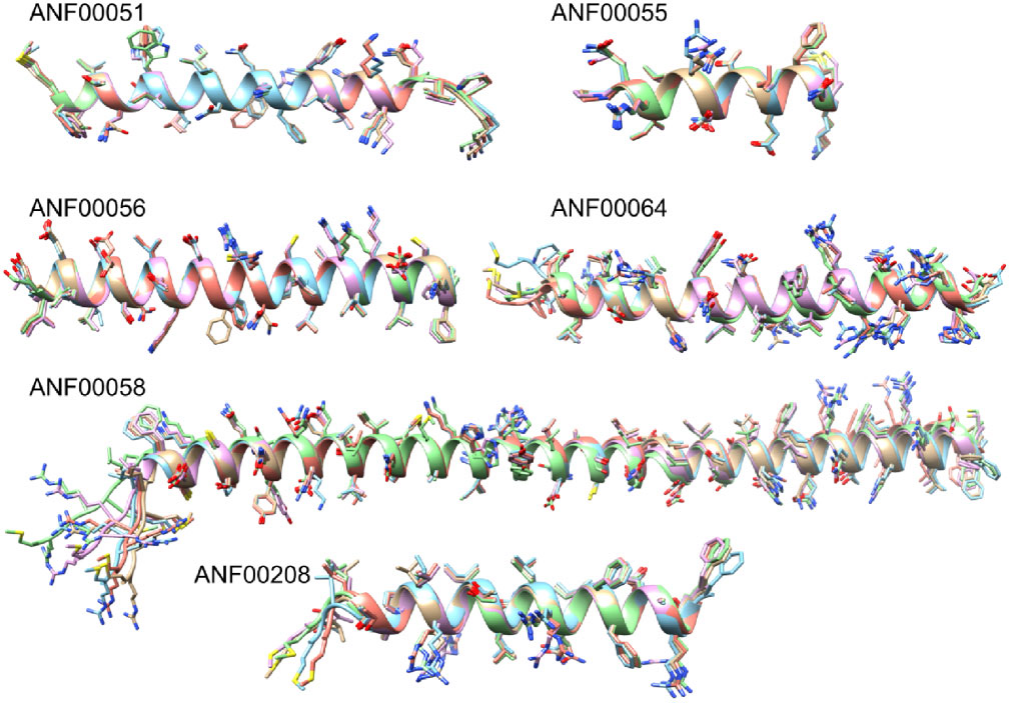
AlphaFold predictions for the representative sequences of the seven AntiFam families <100 amino acids with a mean pLDDT score >80. The five models for each sequence have been superposed and the side chains shown in stick representation

In Figure 3, we present all the examples of sequences <100 amino acids long with a mean pLDDT score >80 (Supplementary Table S3). It is notable that in every case, the sequence is predicted to be composed of a single alpha-helix. The helical structure was also predicted by the secondary structure prediction tool PSIPRED for AntiFam entry ANF00056, ANF00058 and ANF00064 (Buchan and Jones, 2019). The other three AntiFam entries shown in Figure 3 are too short to be predicted by PSIPRED. The five AlphaFold models predicted per sequence superpose very well and in many cases the side chain orientations are identical between the models (Fig. 3).

**Fig. 4.**
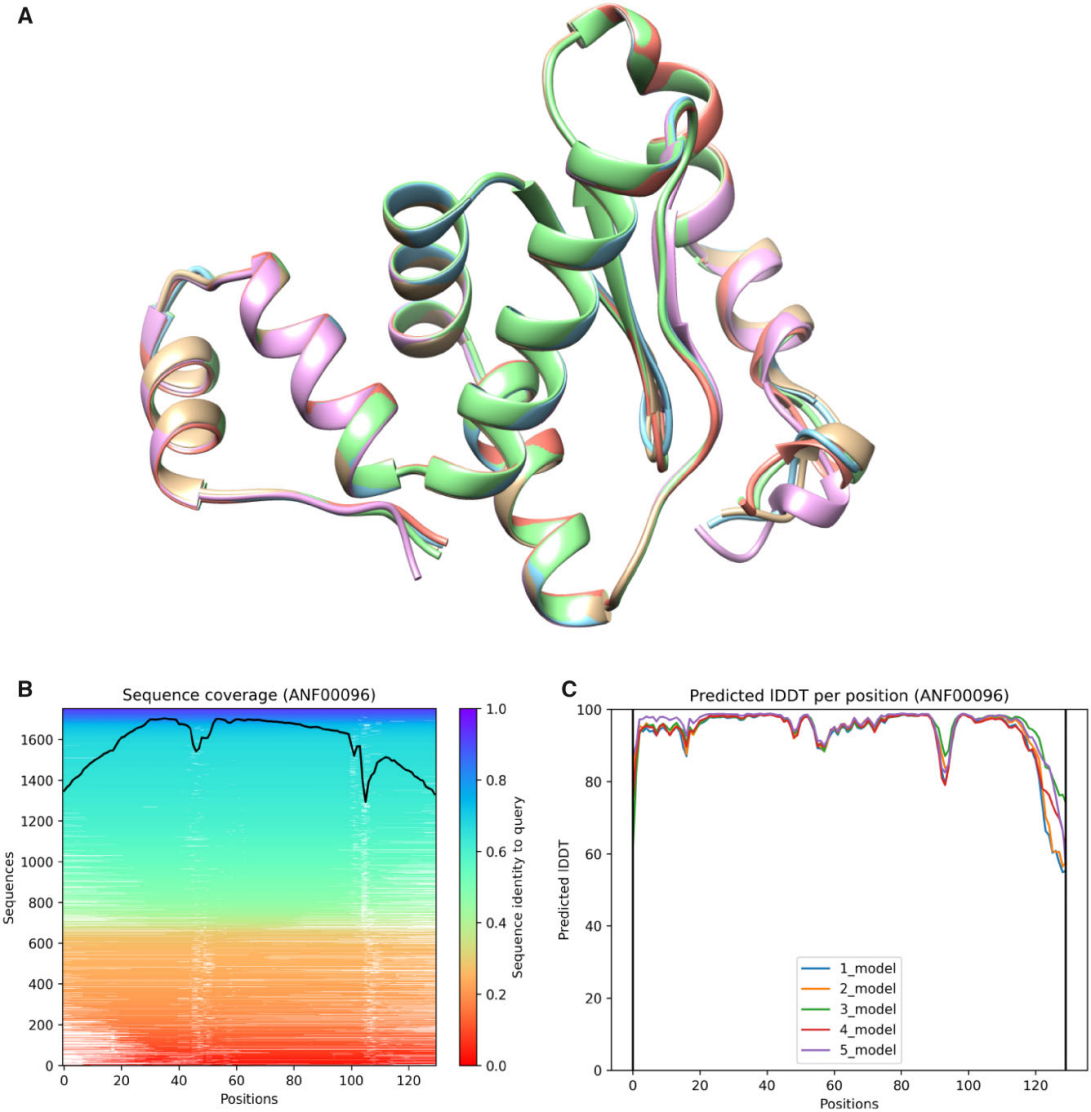
AlphaFold predictions for representative sequence of AntiFam entry ANF00096. (**A**) Superposition of the five AlphaFold predictions. (**B**) Sequence coverage plot showing the number of homologues identified across the representative sequence and colored by the sequence identity of the homologues. (**C**) A plot of the pLDDT score per position for each of the five AlphaFold models predicted

We only identified a single AntiFam representative sequence, for entry ANF00096, where a high-quality structure was predicted of length >100 amino acids (see Fig. 4). Several aspects of sequence and distribution for ANF00096 family open reading frames had independently generated the notion that members of the family are expressed, functional proteins rather than artifacts of genome misinterpretation. In particular, manual inspection of a multiple alignment of ANF00096 family protein translations showed strong conservation of length with few examples of truncation or overhang at either N- or C-terminus, which is often seen in spurious protein translations. In the interior of the alignment, insertions and deletions were common but occurred without shifting the reading frame. The overall amino acid composition seemed typical of globular proteins, rather than showing overrepresentations of Pro (CCN), Gly (GGN), Ala (CGN) and Arg (GCN or AGR) as typical for spurious translations in GC-rich lineages, such as Streptomyces. Most columns of the multiple alignment showed expected patterns of conservative substitution, with strong conservation of hydrophilic, hydrophobic, aromatic, small side chain size or Cys or Pro presence over a large fraction of residue positions. A search using the PaperBLAST resource (Price and Arkin, 2017), allowing BLAST searches against all proteins whose accession numbers or locus tags are cited explicitly in Europe PubMedCentral (Ferguson *et al.*, 2021), found a protein with experimental evidence of gene expression that doubles in response to redox stress (Pires *et al.*, 2020). Lastly, we observed a broad species distribution of member sequences, from Actinobacteria to Cyanobacteria. Some families of ORFs spuriously predicted as proteins, as from tRNA or CRISPR repeat region features, are broadly distributed, but most are highly lineage-specific. All these observations suggested that sequences identified by ANF00096 were likely to consist mostly of genuine functional proteins, but with the caveat that impressions by eye, dependent on curator expertise rather than a computational test, may not be reliable.

Taking all observations into account, we created a Pfam family for the sequences of the AntiFam entry ANF00096 (Pfam: PF20704) and so reinstating this domain family into Pfam.

## 4 Discussion

This study was partly motivated by trying to understand how AlphaFold would perform on non-real protein sequences, to serve as a kind of negative control. Randomly generated sequences would mean that likely no homologous sequences would be found and thus would be an imperfect negative control. The use of AntiFam as a source of sequences means that there are sets of sequences for which we do not believe they are translated in nature and yet we can identify similar sequences in the sequence databases. It is important to note that AlphaFold was not designed or trained to make predictions for spurious or randomly generated protein sequences. A second motivation was to see if we could use AlphaFold to act as a quality control on AntiFam. If AntiFam entries were truly spurious we would not expect them to adopt a well-ordered globular structure.

Our discovery of a correlation between sequence length and mean pLDDT is both an interesting and important one. An outstanding question raised by this work is whether the short AntiFam sequences do indeed adopt the confidently predicted conformation that AlphaFold has produced. Some of the shortest sequences, such as that for ANF00055, are both confidently predicted and have highly consistent structure prediction even down to the side chain orientations. The helical structures predicted by AlphaFold for the entry ANF00056, ANF00058 and ANF00064 were also endorsed by the secondary structure prediction tool PSIPRED. The question whether the short AntiFam sequences indeed fold into helical structures is related to the question of whether randomly generated sequences will adopt a stable structure (Tretyachenko *et al.*, 2017). Prior work in that area suggests that random sequences can adopt regular secondary structures, although perhaps only 5–20% of these sequences adopt a globular structure.

A better distinction between spurious and *bona fide* proteins appears to be achieved by the pTM score, which is below 0.5 for all AntiFam entries, except the likely false positive entry ANF00096. But also the pTM score tends to be increased for shorter compared to longer representative AntiFam sequences and thereby yields no clear separation to real proteins of the manually reviewed SwissProt database for sequences below 100 residues length.

The computational structure determination method can only determine that AntiFams are unlikely to be spurious. It does not confirm the spurious nature of each entry. For example, well known disordered proteins lack a well-defined AlphaFold structure and disordered regions are described to substantially overlap with low confidence score regions (Akdel *et al.*, 2021; Ruff and Pappu, 2021). Therefore, this property cannot be used solely to determine if a protein is likely to be a spurious translation. AntiFam is heavily biased toward eubacterial proteins, with 223 of the 250 AntiFam entries containing eubacterial sequences and only 25 containing eukaryotic sequences. Eubacteria have a far lower proportion of disordered proteins than eukaryotes (Dunker *et al.*, 2000) and thus true protein translations are much more likely to contain ordered globular regions than in eukaryotic proteins. Thus, this methodology, we apply is well suited to AntiFam, but it is likely to be far less effective for eukaryotic sequences.

Overall, we have discovered that AntiFam as expected has almost no entries that show a globular structure thus providing an independent quality assessment for the resource. This work and the use of AlphaFold can be used as a quality control method for existing and candidate AntiFam entries, and has enabled the removal of an erroneously added true protein entry from AntiFam. However, short regions of secondary structure, such as a single alpha-helix should not be taken as the sole evidence that a short peptide sequence is a true translated protein and should be removed from AntiFam.

Initially, we thought that AlphaFold predictions might play a role in the accurate determination of the protein-coding content of a genome. For example, by looking through alternate reading frames looking for confidently predicted structures. However, our results on AntiFam suggest that AlphaFold may be able to distinguish between spurious and true protein-coding genes for longer ORFs, but it is probably not a useful tool for short ORFs. Due to the computationally intensive nature of carrying out AlphaFold predictions, it seems likely that such a method would only be applied to high value reference proteomes to help discover long novel missing ORFs. For the large majority of genomes now being sequenced, high speed prediction is critical for scalability.

In conclusion, the field of identifying spurious proteins has been greatly hindered by a lack of tools to identify them, as well as the difficulty in confidently deciding that a protein is not translated. AlphaFold provides a new and exciting adjunct to the existing techniques in this field. Through the study of spurious proteins with AlphaFold, we have also identified an important correlation between protein length and the confidence prediction of AlphaFold. This result has important implications for interpreting AlphaFold outputs for shorter peptide sequences in both spurious and real protein sequences.

## Data availability

The AlphaFold predictions for representative sequences can be found at the following URL: https://drive.google.com/drive/folders/1u9OocRIAabGQn56GljoG1JTDAxjkY1ro.

The representative AntiFam sequences used in this study as well as the scripts used for the graphs are provided under the following GitHub repo: https://github.com/VivianMonzon/Folding_the_Unfoldable.

## Funding

This work was supported by the Intramural Research Program of the National Library of Medicine, National Institutes of Health (to D.H.H.). V.M. and A.B. were supported by core EMBL funding.

## Conflict of Interest

A.B. is Editor-in-Chief of *Bioinformatics Advances*, but was not involved in the editorial process of this manuscript.

## Supplementary Material

vbab043_Supplementary_Data
