# Supplementary material for: Folding the unfoldable: using AlphaFold to explore spurious proteins
Source: Bioinform Adv. 2022 Jan 9;2(1):vbab043. doi: 10.1093/bioadv/vbab043 (PMC9710616; doi:10.1093/bioadv/vbab043)
Supplement: vbab043_Supplementary_Data [file vbab043_supplementary_data.zip › Supplementary.docx]

**Supplementary**

Table S1: ‘Randomly_created_sequences.xlsx’

Table S2: SwissProt identifiers used in this study. For each sequence length five proteins were selected.

| **Length** | **Sequence 1** | **Sequence 2** | **Sequence 3** | **Sequence 4** | **Sequence 5** |
| --- | --- | --- | --- | --- | --- |
| 10 | P85846 | P85862 | P22690 | P85851 | P21996 |
| 16 | P0DMB3 | P0DTU0 | C0HL49 | Q7LZ48 | P0CI05 |
| 20 | P84269 | P0C823 | P0C826 | P0C818 | C0HJK0 |
| 30 | P83641 | P58452 | A0T0B4 | P82416 | P10294 |
| 40 | A8W3J7 | P69691 | B1WR29 | A5GQT7 | P48117 |
| 50 | P64756 | P77091 | P10436 | A8G2R2 | Q48WA5 |
| 60 | P53507 | Q6HPP0 | O13931 | Q9Y0Y0 | A2RC32 |
| 70 | P48129 | A0PXV4 | E7EKI9 | A9IK87 | Q3MBD4 |
| 80 | P83311 | P0DQM3 | Q5N3E5 | P0C980 | B3DYW5 |
| 90 | Q0TNH5 | Q65UA8 | Q0BJ37 | Q9HU36 | A0RPX5 |
| 100 | Q9A4S4 | A8W3C2 | B4SYZ0 | Q97JL5 | Q11EW7 |
| 120 | Q8XEK5 | P67301 | O21942 | A3PGR0 | Q755X2 |
| 140 | A5WBS6 | A4YCX0 | Q8YW20 | Q2YWK0 | Q0AQB0 |
| 160 | B0VSB7 | Q5PNN8 | A6U0U2 | P0C9K0 | P0CV29 |
| 180 | B1KSL3 | Q2IJ73 | P81477 | Q1CI38 | B2ZCQ3 |
| 200 | O73770 | P39421 | B0KPW7 | Q5UQC7 | Q73AU3 |

Table S3: Additional information regarding the AntiFam sequence entries shown in Figure 2 with a sequence length below 100 residues and an average pLDDT score above 0.8.

| **Antifam entry** | **Length** | **Mean pLDDT score** | **PSIPRED** | **Sequence** |
| --- | --- | --- | --- | --- |
| ANF00051 | 29 | 88.37 | * | MWELTQTWILKINFQKFRYILNKKKQVPK |
| ANF00055 | 16 | 84.53 | * | PQRILRDNTLEIFNKM |
| ANF00056 | 36 | 83.75 | Helical | TILELLESSQITKCDGFCRNYCVLLKISKEL  SCKFF |
| ANF00058 | 80 | 87.86 | Helical | MRRRMLPFDCKCSFQQYQQKDQLLEIQS  VMAVKHINHYARLEQASCDINSSVDPITSS  LHDFIQNRQTFVKVRKLVSFFC |
| ANF00064 | 35 | 80.98 | Helical | MPDALRLSGLRHLYNILNLRAFVGRIRRSR  SIRHE |
| ANF00208 | 25 | 81.46 | * | MTLTAPLRRITLQLRQIFFTEARTF |

***** Sequence too short to be run by PSIPRED
